# 3D genome organization

**DOI:** 10.1038/s41598-022-26725-7

**Published:** 2022-12-22

**Authors:** Asli Silahtaroglu, Joanna M. Bridger, Elissa P. Lei

**Affiliations:** 1 Department of Cellular and Molecular Medicine, Faculty of Health and Medical Sciences, University of Copenhagen, Copenhagen, Denmark; 2 Centre of Genome Engineering and Maintenance, College of Health, Medicine and Life Sciences, Brunel University London, London, UK; 3 Nuclear Organization and Gene Expression Section, Laboratory of Biochemistry and Genetics, National Institute of Diabetes and Digestive and Kidney Diseases, National Institutes of Health, Bethesda, MD USA

**Keywords:** Molecular medicine, Genetics

## Abstract

Our genomes are highly organized spatially in three-dimensions (3D). In interphase nuclei, the genome is anchored and regulated by various nuclear scaffolds and structures, including the nuclear lamina at the nuclear edge, and nucleoli located more internally within the nucleoplasm. Recently, great effort has been made to understand the intricacies of 3D genome organization and its relevance to genomic and nuclear function. Over the years, many concepts, mathematical models, visual and biochemical methods, and analysis pipelines have been presented to study various aspects of this organization in a multidisciplinary manner, such as is also reflected within this collection.

These efforts have led to the discovery of new structures at the chromatin level or improved our understanding of genomic organization at an even higher resolution than before. Initially, individual chromosomes were shown to occupy distinct territories within interphase nuclei using fluorescence in situ hybridization technique^1^. However, in recent years, the development of chromatin conformation capture (3C) based proximity mapping techniques has revealed that the genome is indeed separated into compartments, and furthermore into self-interacting genomic regulatory units termed topologically associating domains (TADs)^2–4^. Further improvements to 3C based methods have allowed for the detection of typical enhancer-promoter loops. Most of these higher resolution approaches are based on averages derived from cell populations in asynchronous cultures. Development of super-resolution microscopy techniques^5^ and advances in new probe technologies, particularly the Oligopaint approach combined with automated sequential hybridization, have led to a better understanding of these proposed chromatin structures at a single cell level with improved resolution^6^.

In this 3D Genome Organization Collection, several groups have presented technical developments that increase the resolution power to interrogate the structure of the genome at a more detailed level. Hirata et al. presented an imputation-free computational method for individually reconstructing the 3D architecture of each chromosome by integrating allele-specific single nucleotide variation data for each chromosome pair into a Hi-C dataset from single diploid cells^7^. Li et al. introduced a chromatin packing domain structure, based on their observations employing chromatin scanning transmission electron microscopy (ChromSTEM), together with DNA-specific staining^8^. The authors used a polymer physics-based algorithm to analyze their data and detected the higher order chromatin packing domains of 60–160 nm in diameter. They concluded that chromatin packing was higher at the nuclear periphery^8^. Parteka-Tojek et al. were able to push the limits of in situ hybridization technologies using Oligopaint probes with interferometric photoactivated localization microscopy and visualized the spatial conformation of a 13 kb chromatin loop with a 120–300 bp genomic resolution and 4–22 nm microscopic precision in X-, Y-, Z-dimensions in human lymphoblastoid cells. The authors suggested that the method could allow for the visualisation of the loop extrusion process by taking snapshots in single cells^9^. Combined with other approaches, this should help to elucidate the connection between chromatin looping and transcription.

A second aspect of this special Collection focuses on the biological consequences of perturbations in cellular 3D organization. Korolev et al. studied the elasticity of the genome after physical expansion and compaction of nuclei. They performed Hi-C and microscopy following either swelling the nuclei under low salt concentration or extending and retracting the chromatin physically. While slight changes were observed in short distance interactions, higher order structures such as loops, TADs compartments, and chromosome territories maintained their global organization. The authors conclude that this robustness of genome topology offers resilience against physiological changes that take place in nuclear morphology^10^.

As more clinical data are accumulated, 3D genome organization is revealing itself as a spatial epigenetic mechanism to fine-tune gene expression. The nuclear lamina acting both as an anchor and a regulatory site plays a fundamental role in this organization. Kychgina et al. studied Hutchinson-Gilford Progeria Syndrome (HGPS), a premature ageing condition caused by aberrant splicing and processing of *LMNA*, leading to the production of a mutant form of lamin A termed Progerin. The authors found that expression of Progerin altered 3D telomere organization and altered the chromatin state and the replication timing of the telomeres. They suggested that Progerin expression increases the contacts between the telomeres themselves and the lamina, which led to replication stress, resulting in shortening of telomere size. This telomere shortening triggered premature growth arrest in cells, leading to premature senescence. The HGPS cells were found to have low nucleoside pools and the addition of dNTPs permitted some rescue of the replication stress^11^. Gallant et al. introduced a high-throughput automated visual assay to detect DNA damage (HiIDDD) based on measurement of 53BP1 and ƴ-H2AX levels. This assay can obtain data from a large number of cells concomitantly, and may be particularly useful in cancer, inflammation, drug development, and ageing studies^12^.

We are still a long way from understanding how 3D genome organization is linked precisely to genome function. A concerted multi-disciplinary effort is needed to develop new tools and computational prediction methods, multi-target chromatin imaging techniques in live-cells, and efficient manipulation methods for 3D genome structures. These efforts should be accompanied by the collection of 3D genome data from different diseased and healthy cells and tissues in humans, as well as a range of model organisms. Our increased knowledge of 3D folding of the genome will lead to a better appreciation of the regulatory potential of the linear genetic sequence. 3D genome organization emerges as a cell type specific epigenetic mechanism and gives us clues about the regulatory effect of the non-coding genome in the 3D context. This understanding will allow for enhanced interpretation of genetic variants and their potential phenotypic effects. Finally, such studies will bring new 3D insights into diagnostics and therapies for different conditions including cancer, developmental diseases, ageing, and related disorders.
